# Supplementary material for: Short-Term Acute Exposure to Wildfire Smoke and Lung Function among Royal Canadian Mounted Police (RCMP) Officers
Source: Int J Environ Res Public Health. 2021 Nov 10;18(22):11787. doi: 10.3390/ijerph182211787 (PMC8618710; doi:10.3390/ijerph182211787)
Supplement: Supplementary file 1 [file ijerph-18-11787-s001.zip › ijerph-1410510-supplementary.pdf]

Short-term acute exposure to wildfire smoke and lung function among Royal Canadian Mounted Police (RCMP) officers

Subhabrata Moitra, Ali Farshchi Tabrizi, Dina Fathy, Samineh Kamravaei, Kawtar Idrissi Machichi, Noushin Miandashti, Linda Henderson, Fadi Khadour, Muhammad T. Naseem, Nicola Murgia, Lyle Melenka, Paige Lacy

List of supplementary Tables and Figures

1. **Supplementary Table S1:** Component (unrotated) loading matrix of the principal component analysis (PCA)
2. **Supplementary Figure S1:** Scree plot of the principal component analysis
3. **Supplementary Figure S2:** Component loading plot (Bi-plot) of the PCA
4. **Supplementary Table S2:** Changes in lung function in association with per interquartile (IQR) change in air pollution index
5. **Supplementary Table S3:** Effect modification by smoking, any airway obstruction, childhood smoke exposure, and any parental lung disease on the association between the air pollution index (per interquartile change) and lung function indices of the RCMP officers.

Supplementary Table S1: Component (unrotated) loading matrix of the principal component analysis (PCA)

Variables	Comp 1	Comp 2	Comp 3	Comp 4	Comp 5	Comp 6	Comp 7
**CO**	0.39	-0.14	-0.41	-0.15	0.04	0.09	-0.79
**CH_4**	0.35	0.62	0.32	0.44	0.15	0.41	0.04
**NO**	0.38	0.38	-0.06	-0.75	-0.38	0.08	0.01
**NO_2**	0.40	-0.14	0.05	0.43	-0.66	-0.40	0.20
**O_3**	0.39	-0.05	0.47	-0.18	0.49	-0.59	-0.11
**SO_2**	0.35	-0.65	0.33	-0.12	0.01	0.55	0.16
**$PM_{2.5}$**	0.38	-0.02	-0.64	0.04	0.40	-0.04	0.54

Values are in $\mu g/m^3$.

Abbreviations: CO: carbon monoxide; CH_4: methane; NO: nitric oxide; NO_2: nitrogen dioxide; O_3: ozone; SO_2: sulphur dioxide; $PM_{2.5}$: particulate matter of aerodynamic diameter ≤2.5μm.

Supplementary Figure S1: Scree plot of the principal component analysis

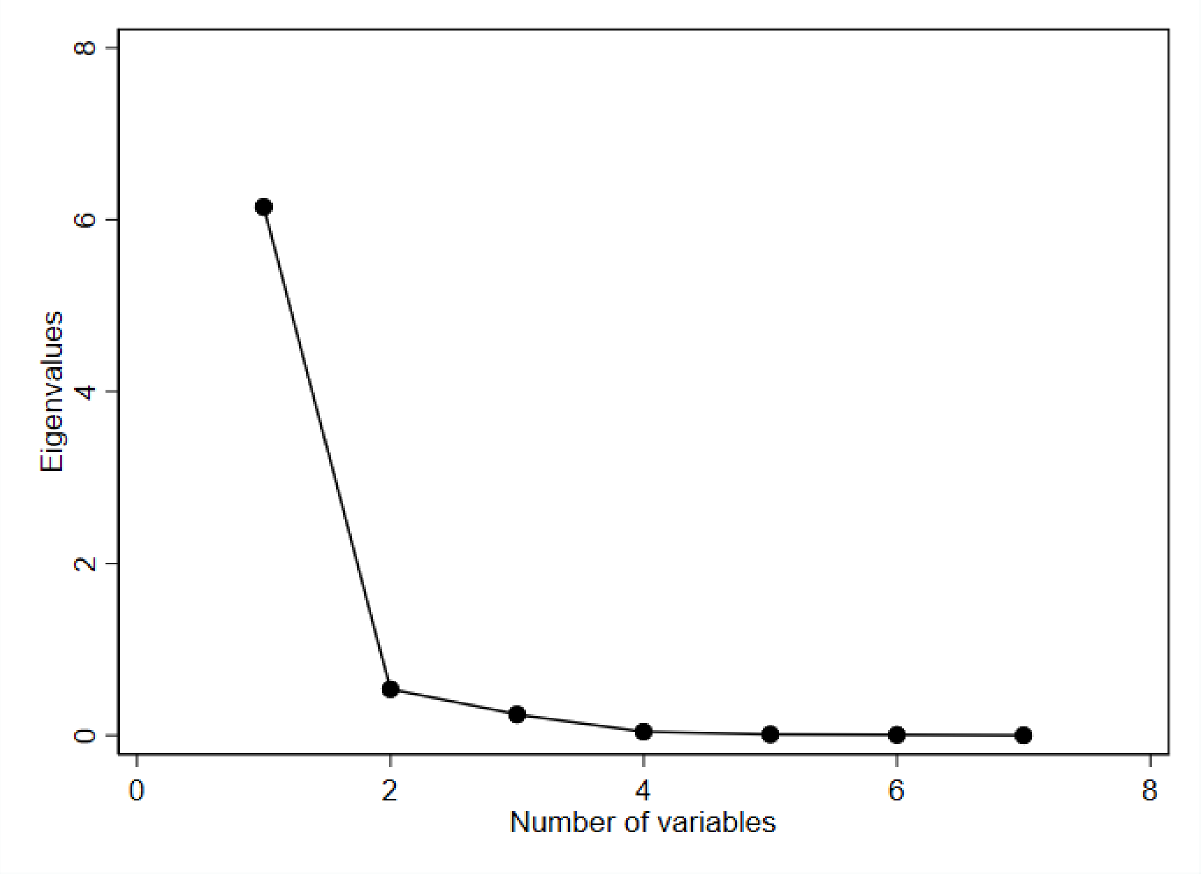

Supplementary Figure S2: Unrotated component loading plot (Bi-plot) of the PCA

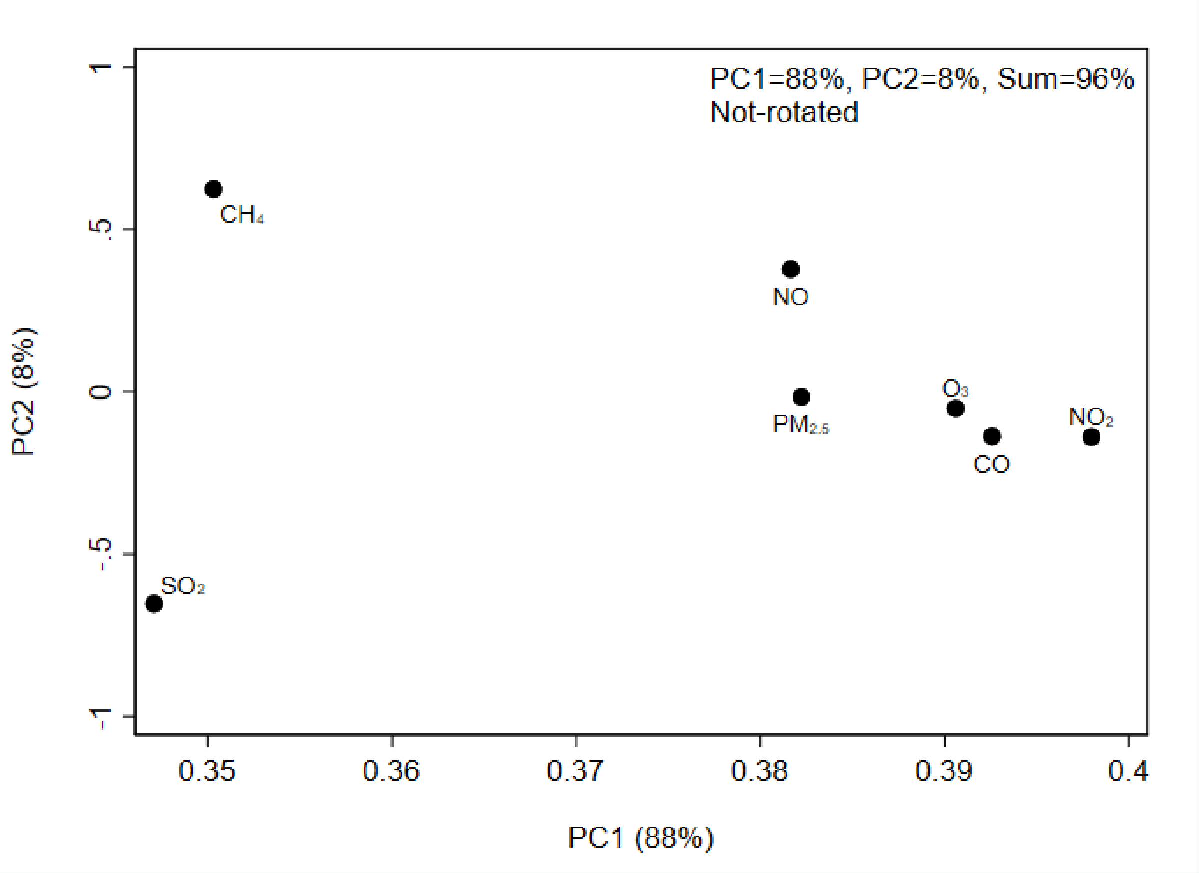

Abbreviations: CO: carbon monoxide; CH_4: methane; NO: nitric oxide; NO_2: nitrogen dioxide; O_3: ozone; SO_2: sulphur dioxide; $PM_{2.5}$: particulate matter of diameter ≤2.5μm.

Supplementary Table S2: Changes in lung function in association with per interquartile (IQR) change in air pollution index

Variables	β (95% CI)		
	**Model-1**	**Model-2**	**Model-3**
FVC (% predicted)	0.36 (-0.80, 1.52)	0.29 (-0.85, 1.43)	0.30 (-0.84, 1.45)
FEV_1 (% predicted)	0.37 (-0.79, 1.53)	0.35 (-0.77, 1.48)	0.36 (-0.77, 1.48)
FEV_1/FVC (%)	-0.20 (-0.75, 0.35)	0.01 (-0.50, 0.52)	0.005 (-0.51, 0.52)
TLC (% predicted)	0.50 (-0.54, 1.54)	0.43 (-0.60, 1.45)	0.43 (-0.60, 1.46)
RV (% predicted)	1.76 (-0.06, 3.57)	1.55 (-0.28, 3.37)	1.56 (-0.26, 3.37)
RV/TLC (%)	0.40 (-0.05, 0.85)	0.28 (-0.12, 0.67)	0.28 (-0.12, 0.67)

Data presented as linear regression coefficient (β) and 95% confidence interval, unless otherwise specified. Coefficients were calculated in respect to per IQR change (μg/m^3) of the principal component (PC). Model-1: unadjusted model; Model-2: adjusted by age, sex, BMI, race, and smoking history. Model-3: Model-2 + PPE use (yes/no).

Abbreviations: BMI: body mass index; FEV_1: forced expiratory volume in 1 second; FVC: forced vital capacity; TLC: total lung capacity; RV: residual volume; PPE: personal protective equipment.

Supplementary Table S3: Effect modification by smoking, any airway obstruction, childhood smoke exposure, and any parental lung disease on the association between the air pollution index (per interquartile change) and lung function indices of the RCMP officers

**Effect modifier**		**FVC (% predicted)**	**FEV_1 (% predicted)**	**FEV_1/FVC (%)**	**TLC (% predicted)**	**RV (% predicted)**	**RV/TLC (%)**
Smoking*	No (N=174)	0.55 (-0.75, 1.85)	0.35 (-0.93, 1.63)	-0.13 (-0.71, 0.45)	0.65 (-0.52, 1.82)	1.82 (-0.18, 3.82)	0.30 (-0.12, 0.71)
	Yes (N=42)	-0.35 (-3.10, 2.39)	1.11 (-1.61, 3.84)	0.76 (-0.40, 1.93)	0.13 (-2.35, 2.60)	2.25 (-3.04, 7.53)	0.63 (-0.60, 1.87)
	$P^{\#}$	0.32	0.95	0.30	0.35	0.56	0.89
Any airway obstruction	No (N=149)	0.03 (-1.27, 1.32)	0.03 (-1.20, 1.25)	-0.13 (-0.60, 0.35)	0.34 (-0.78, 1.47)	1.62 (-0.36, 3.60)	0.30 (-0.14, 0.73)
	Yes (N=66)	1.74 (-1.21, 4.68)	1.61 (-1.37, 4.60)	0.09 (-1.39, 1.57)	1.42 (-1.31, 4.15)	3.23 (-1.43, 7.90)	0.53 (-0.44, 1.50)
	$P^{\#}$	0.22	0.24	0.73	0.35	0.48	0.70
Childhood smoke exposure	No (N=87)	0.93 (-0.78, 2.64)	0.36 (-1.19, 1.91)	-0.46 (-1.12, 0.20)	0.94 (-0.59, 2.47)	**2.71 (0.11, 5.30)**	0.48 (-0.04, 0.99)
	Yes (N=131)	-0.37 (-2.00, 1.24)	0.35 (-1.32, 2.03)	0.50 (-0.27, 1.26)	-0.19 (-1.64, 1.27)	0.16 (-2.49, 2.82)	0.05 (-0.55, 0.65)
	$P^{\#}$	0.26	0.91	0.09	0.26	0.18	0.34
Parental lung disease	No (N=185)	0.30 (-1.11, 1.71)	0.43 (-0.90, 1.76)	0.01 (-0.60, 0.63)	0.60 (-0.68, 1.88)	2.10 (-0.17, 4.38)	0.37 (-0.12, 0.85)
	Yes (N=33)	0.56 (-1.39, 2.51)	0.57 (-1.80, 2.95)	0.11 (-0.93, 1.16)	0.16 (-1.44, 1.75)	-0.03 (-0.293, 2.88)	-0.06 (-0.78, 0.65)
	$P^{\#}$	0.82	0.89	0.83	0.78	0.38	0.42

Data presented as linear regression coefficient (β) and 95% confidence interval, unless otherwise specified. Coefficients were calculated in respect to per IQR change (μg/m^3) of the principal component (PC) adjusting for age, gender, BMI, race, and smoking history (*except for Smoking). $P^{\#}$: p value for interaction. Abbreviations: BMI: body mass index; FEV_1: forced expiratory volume in 1 second; FVC: forced vital capacity; TLC: total lung capacity; RV: residual volume.
